# The Assessment of Parameters Affecting the Quality of Cord Blood by the Appliance of the Annexin V Staining Method and Correlation with CFU Assays

**DOI:** 10.1155/2013/823912

**Published:** 2013-03-07

**Authors:** Teja Falk Radke, David Barbosa, Richard Charles Duggleby, Riccardo Saccardi, Sergio Querol, Gesine Kögler

**Affiliations:** ^1^Institute of Transplantation Diagnostics and Cell Therapeutics, Heinrich Heine University Medical Center, Moorenstra**β**e 5, 40225 Düsseldorf, Germany; ^2^Anthony Nolan Research Institute, Royal Free Hospital, Fleet Road, London NW3 2QG, UK; ^3^Department of Haematology, UCL Cancer Institute, Royal Free Campus, Pond Street, London NW3 2QG, UK; ^4^Cord Blood Bank, Haematology Department, Careggi University Hospital, Via delle Oblate 1, 50141 Firenze, Italy; ^5^Eurocord ARTM, Carré Historique Porte 05, Hôpital Saint-Louis, 1 Avenue Claude Vellefaux, 75010 Paris, France; ^6^Barcelona CBB, Banc de Sang i Teixits, Passeig Taulat 116, 08005 Barcelona, Spain

## Abstract

The assessment of nonviable haematopoietic cells by Annexin V staining method in flow cytometry has recently been published by Duggleby et al. Resulting in a better correlation with the observed colony formation in methylcellulose assays than the standard ISHAGE protocol, it presents a promising method to predict cord blood potency. Herein, we applied this method for examining the parameters during processing which potentially could affect cord blood viability. We could verify that the current standards regarding time and temperature are sufficient, since no significant difference was observed within 48 hours or in storage at 4°C up to 26°C. However, the addition of DMSO for cryopreservation alone leads to an inevitable increase in nonviable haematopoietic stem cells from initially 14.8% ± 4.3% to at least 30.6% ± 5.5%. Furthermore, CFU-assays with varied seeding density were performed in order to evaluate the applicability as a quantitative method. The results revealed that only in a narrow range reproducible clonogenic efficiency (ClonE) could be assessed, giving at least a semiquantitative estimation. We conclude that both Annexin V staining method and CFU-assays with defined seeding density are reliable means leading to a better prediction of the final potency. Especially Annexin V, due to its fast readout, is a practical tool for examining and optimising specific steps in processing, while CFU-assays add a functional confirmation.

## 1. Introduction

Since its first application in 1989 by Gluckman et al. [1], transplant using cord blood (CB) as an alternative stem cell source to bone marrow has been well established in clinical practice for the treatment of blood-related diseases. As of today, the enumeration of haematopoietic stem cells (HSCs) by detecting the expression of the surface marker CD34 in flow cytometry following the protocol of the International Society of Hematotherapy and Graft Engineering (ISHAGE) is the most common used technique to predict the potential quality of a unit [2]. Although this analysis allows an immediate readout and the amount of infused CD34^+^ cells in most cases correlates well with the chances of engraftment in the patients [3], exceptions occur in which the ability of the transplant to reconstitute the patients, immune system, the so-called potency, is lower than expected and might result in failure of engraftment [4]. This might rely on different composition of the unit in terms of short-term and long-term haematopoietic stem/progenitor cells, with only the later being responsible for permanent reconstitution of the recipient. Gentry et al. showed that the aldehyde dehydrogenase (ALDH) activity is lower in short-term progenitors and therefore permits the discrimination of these cell types [5].

The influence of the overall quality of the units cannot be discounted, however. This is especially true for cord blood units, which are usually cryopreserved prior to application; a potential loss of function and induction of necrosis and apoptosis due to the freezing/thawing procedure has to be taken into consideration. In this case, simple assessment of HSC by flow cytometric analysis of CD34^+^ cells will lead to overestimating the real potency of the transplant [6–8]. Indeed, CB transplantation is characterized by a slower engraftment kinetics as compared to other SC sources, and overall 10% to 20% of patients are reported to experience an engraftment failure [9, 10]. Therefore, a set of feasible quality controls aimed to assess that the engraftment potential is highly desirable in this clinical setting.

The most common functional in vitro assay currently available for prediction of engraftment is the so-called colony-forming unit (CFU) assay in which cells are cultivated in a semiviscous methyl cellulose media enriched with various haematopoietic growth factors [11]. While this assay gives a much better correlation to the potency [12], its runtime of 7–14 days makes it unsuitable for situations requiring fast decision. 

Recently, Duggleby et al. [13] demonstrated that the flow cytometric assessment of necrotic and early apoptotic cells by staining against 7-aminoactinomycin (7-AAD) and Annexin V (AnnV), respectively, is a feasible method for predicting CFU results. While 7-AAD [14, 15] penetrates only the nonintact membranes of dead cells, Annexin V [16, 17] stains phosphatidylserine which is translocated to the outer membrane in apoptotic cells.

In this paper, we reevaluated and extended this approach by testing the impact of different method- or process-related factors on viability assessment. 

Firstly, the ratio of seeded CD34^+^ cells to colonies observed in the CFU assay was analysed. Commonly, constant sample volumes of cord blood are applied, but the content of haematopoietic cells varies; hence, this might lead to differences in HSC density up to a factor of 10. The ratio of HSC to CFU is often referred to as clonogenic efficiency (ClonE) and regarded as a reliable indicator of potency [18]; therefore, we analysed whether the density of viable HSC seeded affects this ratio by factors such as contact inhibition or nutrient limitation.

Secondly, the influence of storage time and temperature prior to processing was examined. For clinical use, the collection, transport to processing facility, and processing and cryopreservation of the cord blood have to occur within a maximum of 48 hours after birth and temperature must not exceed 22 ± 4°C. In this publication, percentage of dead and apoptotic cells over time was analysed at room temperature and at 4°C, respectively, for up to 72 h. In addition, the effect of long-time exposure to higher temperatures (26°C and 37°C) was assessed.

Thirdly, the level of induction of apoptosis attributed to the addition of the cryoprotectant dimethyl sulphoxide (DMSO) was determined. DMSO is utilised by most banks, and although different publications state that it does not have a direct toxic effect on haematopoietic cells [19–22], a damaging effect due to osmotic shock has been postulated. To overcome this problem, a protocol published by Rubinstein et al. in 1995 [23], with the addition of DMSO for up to 15 minutes and in a final concentration of 10%, is well established. Here, we examined how immediate addition of DMSO does affect apoptosis in CD34^+^ cells in comparison to the slow increase of DMSO concentration over time.

## 2. Material and Methods

### 2.1. Cord Collection, Cryopreservation, and Thawing

Cord blood units (CBUs) were collected and provided by cooperating hospitals and processed at the José Carreras Cord Blood Bank (Düsseldorf, Germany). Processing and cryopreservation was performed within 48 hours after birth with donations not suitable for clinical banking and with informed consent of the mother. To standardize the units involved in this study, the criterion for usage was a minimum in total nucleated cell number (TNC) of 1.1–1.5 × 10^9^. The preparation was performed by volume reduction (Sepax II, Biosafe, Eysins, Switzerland) to 24 mL and the addition of 8 mL of freezing solution consisting of 40% dimethyl sulphoxide (DMSO; CryoSure DMSO, WAK-Chemie Medical, Steinbach, Germany) and 60% Dextran 40 (Delta-Dex 40, DeltaSelect, Dreieich, Germany; final concentration in bag: 10% DMSO, 15% Dextran 40). Cryopreservation was conducted by an automated freezer (Planer 560-16, Messer Group, Bad Soden, Germany), and CBUs were stored in nitrogen-filled tanks in the liquid phase (−196°C). Thawing was carried out according to the Düsseldorf cord blood bank protocol, in compliance with an adapted protocol by Rubinstein et al. [23]. Briefly, the CBUs were thawed using a 37°C water bath and diluted 1 : 3 with the NYCBB washing solution containing 50% dextran 40 (DeltaSelect), 12.5% human serum albumin (HSA 20%; Octalbin, Octapharma, Langenfeld, Germany), and 37.5% phosphate buffered saline (PBS; Dulbecco's PBS, PAA Laboratories, Pasching, Austria).

### 2.2. Assessment of CD34^+^ Cells

CD34^+^ cell numbers were assessed by the double platform-based standard ISHAGE method (International Society of Hematotherapy and Graft Engineering). Therefore, cells were labelled by adding a staining cocktail consisting of CD34-PE (phycoerythrin) and CD45-FITC (fluorescein isothiocyanate) antibodies (BD Biosciences, San Jose, CA, USA) and 7-AAD (Beckman Coulter, Marseille, France) as dye marker (each at an equal volume) and by incubating for 30 minutes at 4°C in the dark. After the addition of 2 mL of lyse buffer (Versa Lyse, BC Beckman Coulter, Marseille, France), an additional 15 minutes of incubation in the dark followed. The assessment of cells was performed on a flow cytometer (FACSCanto, BD Biosciences) within 1 hour using the FACSDiva software (BD Biosciences). Total CD34^+^ cell number was calculated by multiplying the percentage of CD34^+^ cells assessed by the flow cytometer and the white blood cell (WBC) concentration measured by an automated haematology analyser (CELL-DYN Ruby, Abbott Diagnostics, Wiesbaden, Germany) in relation to the total volume of the CBU (fresh: 24 mL, thawed: 29 mL).

### 2.3. Annexin V Assessment Method of Viable CD34^+^ Cells

Sample labelling was performed according to Duggleby et al. [13]. Firstly, 0.6 × 10^6^ cells per sample were stained with CD34-PE and CD45-APC/Cy7 (allophycocyanin, BD Biosciences, San Jose, CA, USA) antibodies in a round-bottom tube (BD Falcon) and incubated for 15 minutes at room temperature in the dark. After lysis, by the addition of 2 mL Lyse buffer and the incubation for 10 minutes (4°C), samples were centrifuged for 7 min (500 g, 4°C) and resuspended in 500 *μ*L Annexin V binding buffer (BD Pharmingen). 3.1 *μ*L of Annexin V-FITC (BD Pharmingen) and 5 *μ*L of 7-AAD were added and incubated for at least 10 minutes at room temperature before flow cytometric measurement was performed. Total viable CD34^+^ cells were calculated by using the AnnV-assessed percentage of viable CD34^+^ cells and the standard ISHAGE-assessed total CD34^+^-cell number as follows:

(1) Total  viable  CD34+cells   =total  CD34+ cells∗[%  Annexin  Vnegative cells].

### 2.4. Colony-Forming Unit Assays and Cloning Efficiency

In order to detect the impact of the cell density on the cloning efficiency (ClonE), colony-forming unit (CFU) assays were performed. Sample volumes (0.5–4.0 *μ*L) were added to a total of 1 mL in semisolid methylcellulose medium (MethoCult H4434, Stem Cell Technologies, USA), after volume reduction and after thawing, respectively. 3∗250 *μ*L was seeded on 24-well plates and incubated at 37°C and 5% CO_2_ in humidified atmosphere.

Cultures were scored on day 14 by microscopic examination by counting distinct colonies of more than 50 cells. For the estimation of the clonal efficiency (ClonE), the total amount of CFU was correlated to the amount of CD34^+^ cells seeded.

### 2.5. Kinetics of DMSO-Induced Apoptosis

24 mL of nonreduced CB was transferred to 50 mL tubes and cooled down to 4°C. WBC concentration was assessed by a haematology analyser, and the volume for 0.6 × 10^6^ cells per sample was calculated. For each CB (*n* = 3), 8 mL of precooled freezing solution (60% Dextran-40 and 40% DMSO) was prepared. 

Two methods of addition were performed in parallel:

- in accordance with Düsseldorf bank's standard operative procedure, the freezing solution was added with a rate of 1 mL/min, reaching a final concentration of 10% after 7 min “progressive addition”;
- addition of the 8 mL freezing solution at once “direct addition”.

After adding the first mL (progressive addition) and after adding the complete freezing solution (direct addition), respectively, samples were taken from both setups during a period of overall 20 minutes (app. 60–70 *μ*L; interval 2–5 minutes) and immediately transferred to 2 mL PBS (PAA Laboratories) to stop the exposure to the concentrated DMSO (WAK-Chemie Medical) by dilution. Samples were centrifuged and prepared for Annexin V assessment as described previously.

## 3. Results

### 3.1. Flow Cytometric Analysis of Fresh versus Thawed Cord Blood Applying Standard ISHAGE and Annexin V Protocols and Correlation with Colony-Forming Units

**Figure 1:**
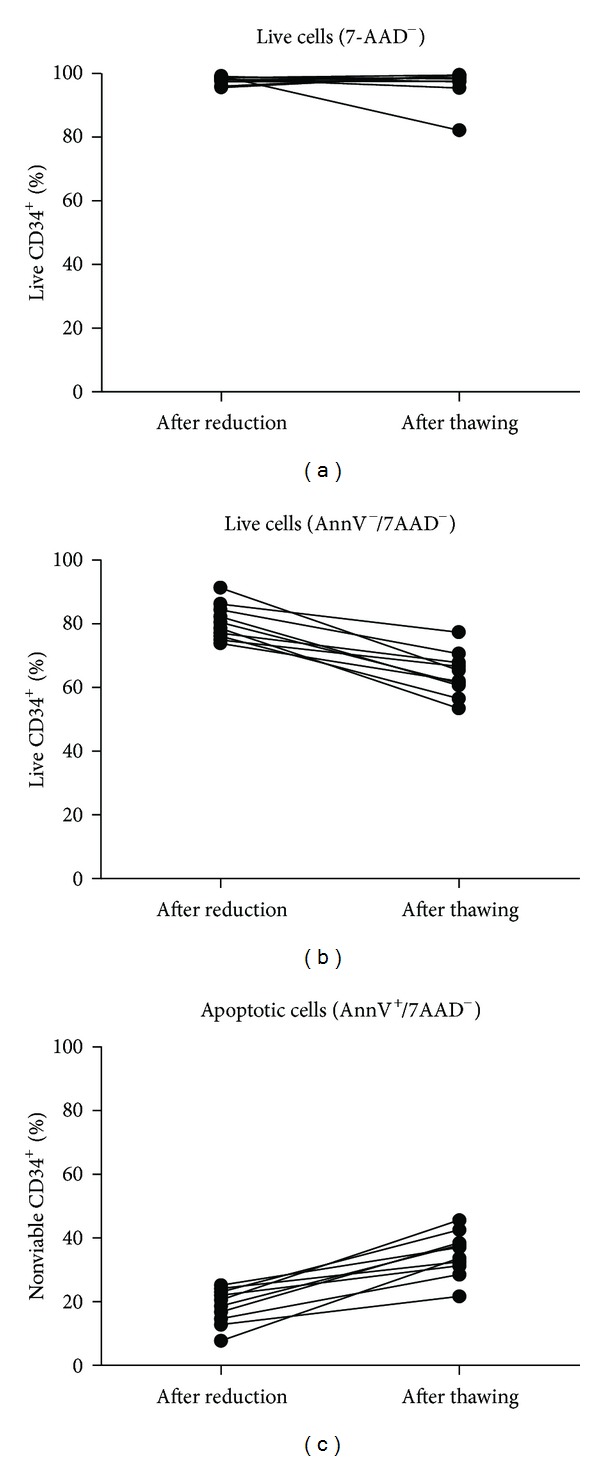
(a) Staining with solely 7-AAD, as in the ISHAGE protocol, showed only low amounts of dead CD34^+^ cells following the volume reduction as well as thawing. ((b), (c)) Combination of 7-AAD with Annexin V revealed already remarkable amounts of apoptotic cells in processed samples ahead of cryopreservation which increases furthermore after thawing. Only cells negative for 7-AAD as well as Annexin V could be regarded as fully viable.

Samples of cord blood units (*n* = 10) were measured directly after volume reduction and after cryopreservation (>7d), respectively. As depicted in Figure 1, we confirmed the results of Duggleby et al. that the processing affects viability of CD34^+^ cells, an effect that is not assessable by the standard ISHAGE protocol.

Although cells other than HSC, predominantly granulocytes, were affected much stronger by the procedure, the results clearly demonstrate an already initial amount of apoptotic CD34^+^ cells of 19% ± 5.6% after volume reduction. After cryopreservation and thawing, this value increased to 36% ±  7%, rendering approximately one-third of the cells potentially unsuitable for transplantation purposes.

**Figure 2:**
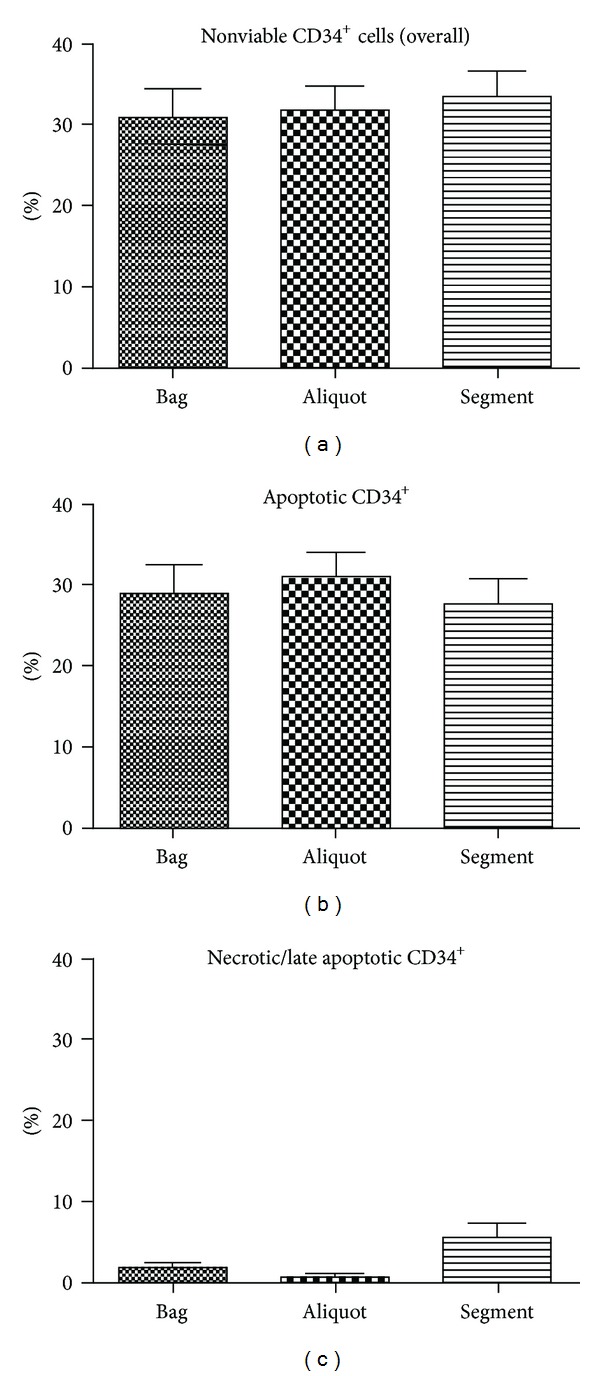
Comparison of Annexin V-determined viability in CD34^+^ cells after thawing between bag, aliquot, and segment. Viability of CD34^+^ cells after thawing (*n* = 5) was assessed, and the resulting necrotic and apoptotic percentages were summed up to obtain nonviable HSC percentage (a). Additionally, apoptotic (b) or necrotic percentages (c) were compared, respectively.

Regarding nonviable cells, no significant differences were observed between bag, aliquot, and segment (*P* = 0,0934, one-way ANOVA test). However, as depicted in Figure 2, the percentages of necrotic/late apoptotic as well as early apoptotic cells differed (*P* = 0.0085 and *P* = 0.0394). With the segment presenting the highest percentage, the aliquot obtained the least necrotic HSC, while values in the bag remained mediocre. Considering the differences in apoptotic percentages, the inverse results were displayed (segment lowest, aliquot highest percentage).

To verify if this discrepancy between the ISHAGE and the Annexin V methods does affect the outcomes of colony-forming unit assays, cells were seeded in semiviscous methyl cellulose either after volume reduction (3.0 *μ*L/mL) or after thawing (3.5 *μ*L/mL; *n* = 10; mean concentration 76.6 ± 37.6 fresh and 83.8 ± 38.2 thawed CD34^+^ cells/*μ*L). As listed in Table 1, both methods lead to similar results and were equally reliable in predicting the final CFU count in “fresh” samples directly after processing. However, the assessment of live, nonapoptotic cells leads to better correlation with colony-forming capacity after thawing.

This correlation of seeded viable CD34^+^ cells and colony formation was compared, resulting in similarly good correlations in fresh (*r* = 0.81 versus *r* = 0.81) and thawed (*r* = 0.86 versus *r* = 0.88) samples for total HSC (ISHAGE) versus viable/nonapoptotic HSC (Annexin V), respectively.

Usually, such a comparison is performed by a normal linear regression to assess the quality of each method. In this way, the data are used to get a hypothetical function.However, it can be argued that a perfect fit to a specific data sample set may define a relationship which deviates from the real relationship through a combination of experimental error, sample distribution, and sample size. 

Whilst the true relationship is unknown, we hypothesise that it is linear and, as anidealisedmathematical model, that each viable CD34^+^ cell can give rise to a haematopoietic cluster.Therefore, a ratio (clonal efficiency, ClonE) between total seeded CD34^+^ cells and formed colonies of 1 : 1 is the maximum reasonable value, giving the slope for the function of *Y* = 1∗*X*. It should be noted that this is the “ideal” case as the CD34^+^ population is heterogeneous and consisting of HSC as well as committed progenitor cells, and therefore the real ratio might be lower. 

**Figure 3:**
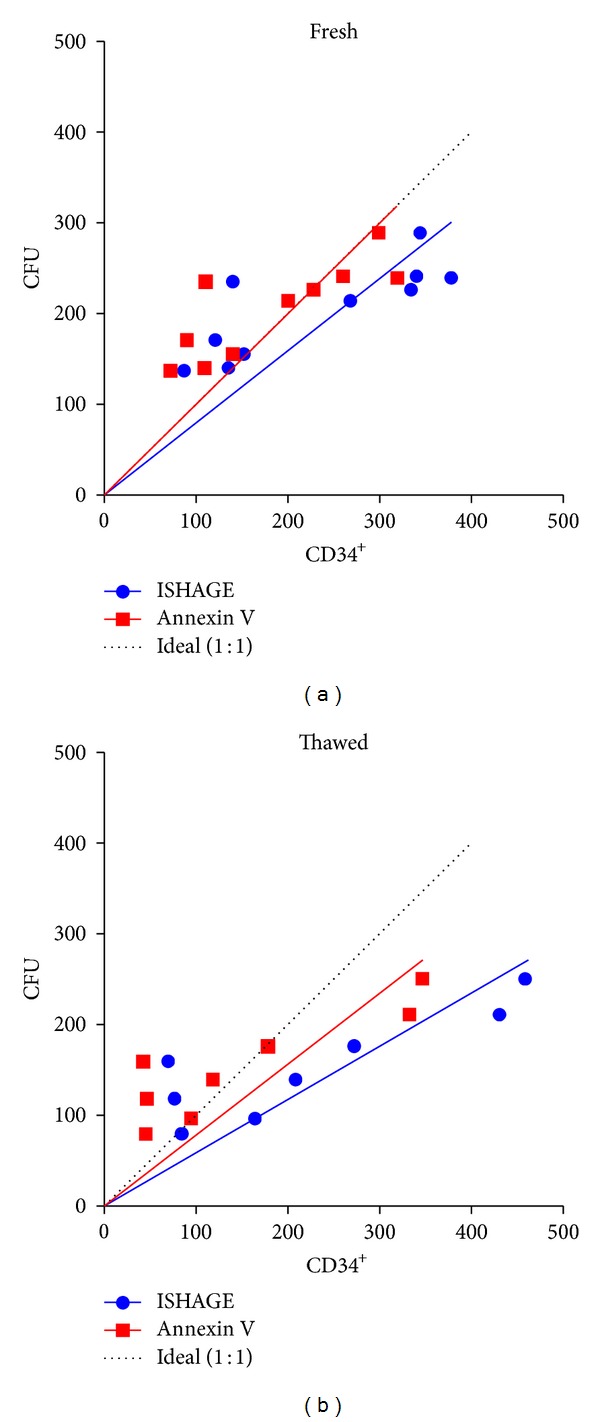
CFU observed versus the amount of CD34^+^ cells seeded assessed by different methods. Following the linear regression, Annexin V-based analysis (red squares) resulted in values closer to the theoretical expected results (dotted line) than those of the standard ISHAGE-protocol (blue circles) either after volume reduction or after thawing.

This idealised linear function was then used as a reference, and following the linear regression (free slope, forced through origin), it gets obvious that the results with Annexin V are closer to this hypothetic line already by graphic display (Figure 3).

For fresh volume-reduced samples (*n* = 10), the ISHAGE protocol gives a slope of 0.7931 (confidence interval 0.6204 to 0.9658), while Annexin V-based measurement results in a regression nearly perfectly matching with the postulated idealised function with a slope of 0.9957 (confidence interval 0.7773 to 1.214).

When analysing the samples after thawing, both methods showed a higher deviation from the hypothetical function with Annexin V displaying a slope of 0.7823 (confidence interval 0.4943 to 1.070) and ISHAGE resulting in a slope of 0.5783 (confidence interval 0.3999 to 0.7566).

It is to note that by both methods, in fresh as well as in thawed samples a tendency for higher CFU to CD34-ratios was observed at lower seeding densities and vice versa. Since it stands to reason that these values are affected by either dilution errors and/or the seeding density, further investigations on this observation were performed.

### 3.2. The Seeding Density of CD34^+^ Cells Critically Influences the CFU Assay

To test if seeding density affects the outcomes of CFU assays, titration arrays were performed in additional experiments. In these experiments, 0.5, 1, 2, 3, and 4 *μ*L of sample volume were seeded per mL of the according media, respectively, and the corresponding total amount of CD34^+^ cells was calculated by CELL-DYN/Flow cytometry.

**Figure 4:**
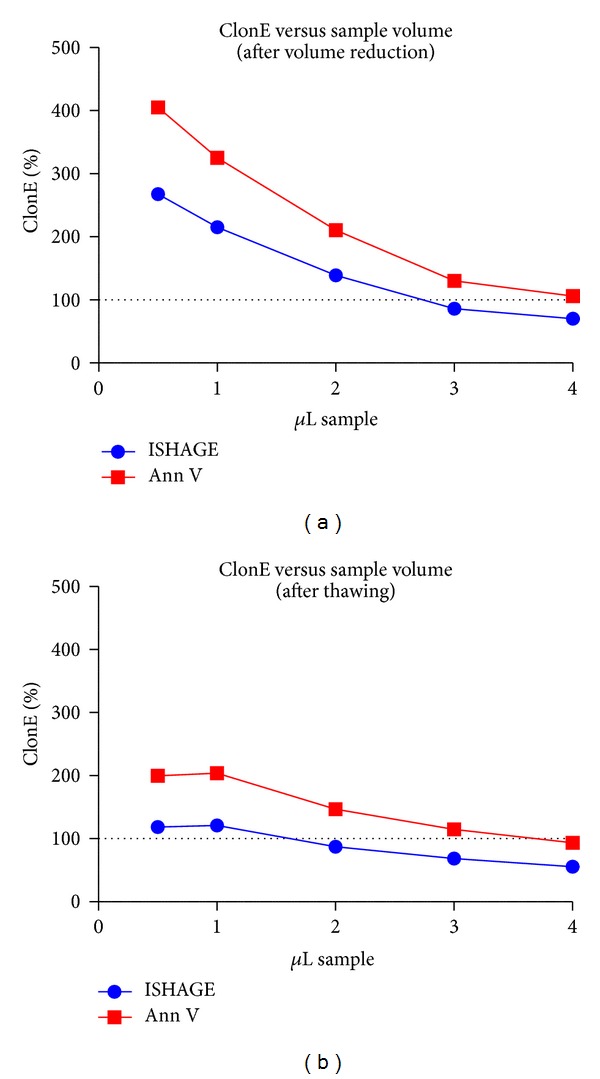
The relation between the concentrations of CD34^+^ cells and the resulting colonies. Seeding low volumes, the clonal efficiency ClonE was as high as 400% or 200% after volume reduction (a) and after thawing (b), respectively. At higher volumes, by representing a higher CD34-seeding density, the results approached boundary limits of nearly 100% (Annexin V) or less (ISHAGE).

The results verified that the observed ratio CFU/CD34^+^ is variable and depending on the volume seeded, ranging from approximately 4 : 1 to 1 : 2 as shown in Figure 4. 

We hypothesised that this might be related to the seeding density, since cell concentration varies between CBUs by factor 4 (ranging from 29 to 115 CD34^+^ cells per *μ*L). Therefore, a detailed evaluation was performed on cord blood units (*n* = 10). These were seeded in the same volumes (0.5 to 4.0 *μ*L sample in 1.0 mL CFU media) but at different CD34^+^ cell concentrations, resulting in a range of 15 to 524 total CD34^+^ cells seeded per mL. 

**Figure 5:**
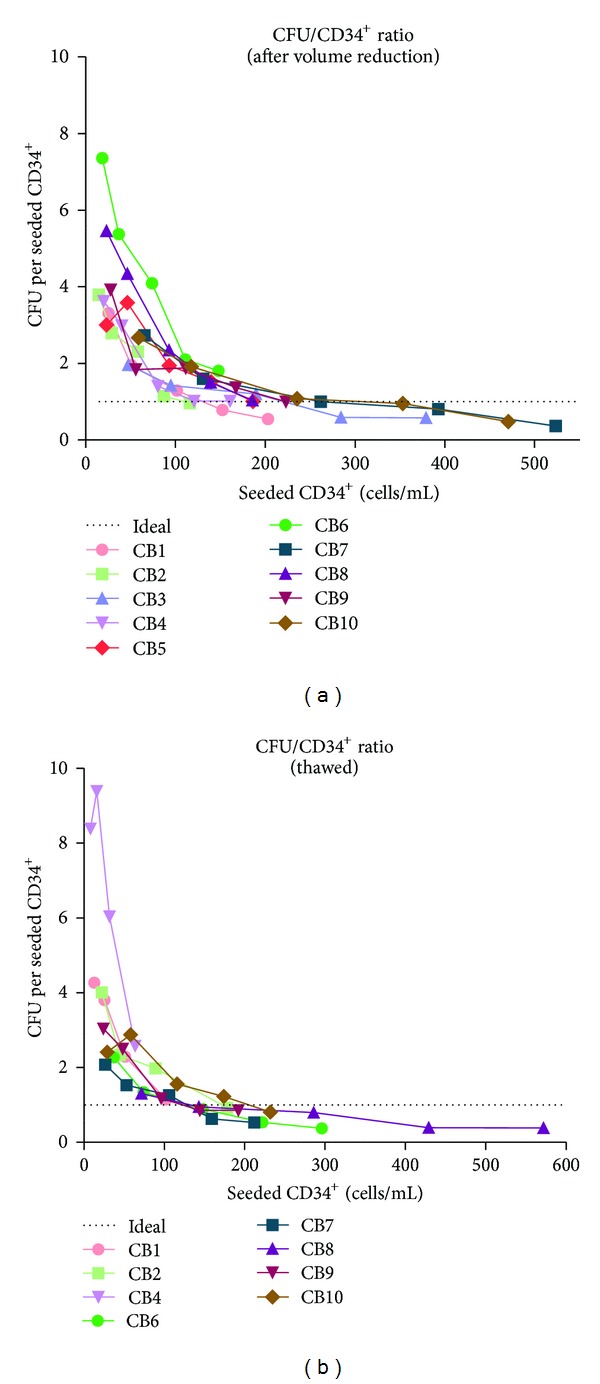
The reproducibility of ClonE for different samples is dependent on seeding density. When less than 100 CD34^+^ cells were seeded per mL, the resulting ratios were extraordinarily high and varied stronger than those with higher seeding density. Only in the range from 100 to 200 CD34^+^/mL, constant ratios were observed close to the theoretically expected value of 1 : 1 (dotted line), while higher densities lead to lower ClonE values, probably due to limiting effects as well as impaired countability.

A strong dependence of ClonE on the seeding density was observed for all the tested CBs, resulting in increased values when seeding less than 150 CD34^+^ cells (Figure 5).

This effect was expected, since especially low densities lead to increased dilution errors, while high density results in colonies overgrowing each other, nutrient limitation, or contact inhibition. Both effects result in increased variations and nearly nonreproducible values for different cord bloods.

However, the data suggested that there is a range in which ClonE is less affected and gives a nearly linear ratio, not only within one sample but also between different CBUs. Moreover, different cord blood units result in a similar ratio, although the concentration of HSC was different. This leads to the assumption that cells other than CD34^+^ are not affecting the colony formation in a significant way. 

### 3.3. The Impact of Storage Time and Temperature on Cell Quality

Only in rare cases, a donated cord blood can be processed in less than 12 hours. On average, the delay between birth and final freezing at the José Carreras Stem Cell Bank is 30 ± 8 hours, while CBUs older than 48 hours are discarded. 

By applying 7-AAD/Annexin V staining method, we examined how the amount of apoptotic CD34^+^ cells changed over time. Additionally, we assessed whether storage at room temperature (RT) is sufficient in maintaining cell quality for up to 72 h and compared it to storage at 4°C as well as 26°C and 37°C, respectively.

**Figure 6:**
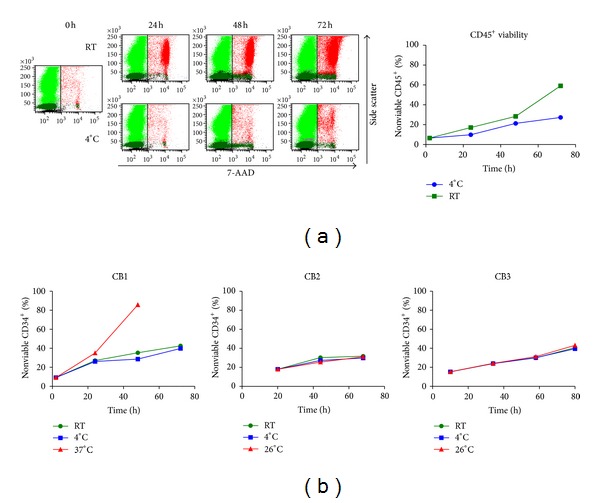
(a) Total leukocytes showed lower viability when stored at room temperature in comparison to 4°C. (b) Regarding CD34^+^ cells, results revealed no difference between storage at 4°C, room temperature and 26°C; only storage at 37°C did drastically increase amount of nonviable cells (exemplarily shown in CB1; red triangles), making the according samples unsuitable for flow cytometric analysis after more than 60 hours.

Figure 6 displays the development of an exemplary CB over time for 4°C and room temperature. Although a difference in the loss of viable leukocytes is obvious (Figure 6(a)), CD34^+^ cells did not reveal a remarkable difference (Figure 6(b)). Additionally, 37°C and 26°C were tested as the highest temperatures on one or two CBUs, respectively.

Already in the initial measurements (11 ± 7 h), a proportion of 14.2% ± 4.5% apoptotic CD34^+^ cells and 25.1% ± 5.9% leukocytes can be observed, and necrotic cells were detectable only in low percentages (0% necrotic CD34^+^ cells; 2.6% ± 2.3% necrotic leukocytes).

Within 36 to 44 hours, the amount of total living leukocytes decreased to 49.4% ± 3.4% at RT, but only to 61.0% ± 6.1% at 4°C. Regarding the percentage of viable CD34^+^ cells, no significant difference between storage at 4°C and storage at room temperature was observed. In both settings, no necrotic cells were detectable for up to 72 h, while apoptotic cells increased to 38.3% ± 4.7% and 36.4% ± 4.6% (*P* = 0.0467).

Even-long-term exposure to 26°C did not affect the kinetics of apoptosis, while in contrast, an ambient temperature of 37°C resulted not only in morphological changes of the cells (as already detected in the CELL-DYN automated haematology analyser) but also in increased amounts of apoptotic and necrotic cells.

### 3.4. The Influence of Dimethyl Sulphoxide on the Percentage of Apoptotic Cells during the Freezing Procedure

Since a significant decrease of viable CD34^+^ cells was observed after freezing and thawing, it was assessed in which step this loss occurs. DMSO, as cryoprotectant, acts through permeabilizing the cell membrane and can, especially at temperatures above 4°C, be cytotoxic over time. According to the protocol published by Rubinstein et al. [23], it should be added slowly (up to a final concentration of 10%; samples are kept at 4°C at all times) in order to prevent an osmotic shock to the cells. However, most publications assessing the impact of DMSO focussed on cell membrane stability after complete freezing and thawing procedure. Therefore, we analysed whether the exposure to DMSO prior to freezing already influences the quality of the cells by applying the Annexin V staining method, which is much more sensitive regarding the viability.

**Figure 7:**
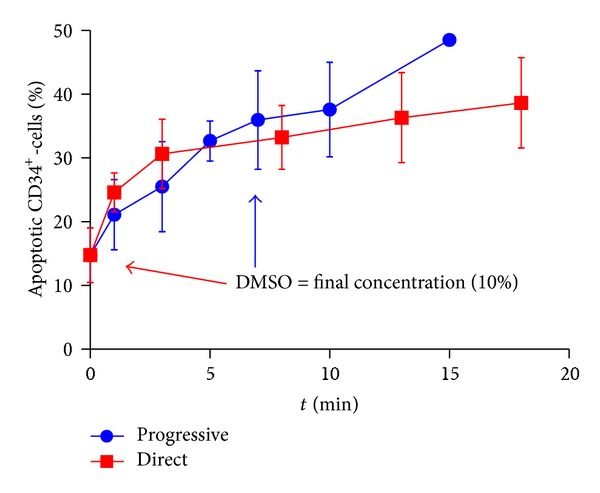
The impact of DMSO on apoptosis of CD34^+^ cells after either progressive (blue circles) or direct (red squares) addition; cord blood units (*n* = 3). Progressive addition leads to a constant loss of viability over time, while direct addition of DMSO resulted in an increased initial loss but slower progression of apoptosis.

As documented in Figure 7, the addition of DMSO does lead to an induction of apoptosis (from initially 14.8% ± 4.3% up to 38.1% ± 6.5%; *n* = 3).

However, the results also reveal that a large fraction of apoptotic cells has already appeared in the initial phase (before the first time point). Further exposure only slightly increased the Annexin V^+^ fraction over time.

Interestingly, when adding DMSO solution without delay, no significant higher percentage of Annexin V-positive cells could be observed in comparison to the progressive addition 3 minutes after reaching the final concentration (30.6% ± 5.5% and 37.6% ± 7.4%; *P* = 0.4372; *n* = 3). On the contrary, after an immediate loss of viable cells, further induction of apoptosis advances slowly (~10% within 20 minutes) in comparison to the progressive addition of DMSO.

## 4. Discussion

The outcome of this study indicates that Annexin V assessment can be reproducible and stable in every part of CBU (bag, aliquot, and segment). 

Handling problems while thawing such as faster thawing and longer exposure to DMSO due to the delayed dilution potentially induced the higher necrotic percentages observed in the segments. Nevertheless, discrepancies in necrotic CD34^+^ cells did not affect the overall nonviable percentages, since apoptotic and necrotic values seem to act reciprocally and compensatory. 

Despite this, the results do not differ significantly in regard to viable cells, but the handling with segments is more disadvantageous for cell viability; the usage of aliquots for viability assessment via the Annexin V staining method would, therefore, be favourable. The presented data also verify that assessment of apoptotic cells via Annexin V staining method, as proposed by Duggleby et al. [13], leads to an improved conformability with the theoretically expected colony formation in vitro.

In comparison to the standard ISHAGE protocol, the Annexin V method resulted in similar good correlations between colony-forming units and CD34^+^ cells seeded. However, Annexin V demonstrated a better matching with the theoretically expected colony formation (assuming a 1 : 1-ration of CD34^+^ cells and CFU). It, therefore, can be postulated that it is more stable and predictive for samples with unusual high amounts of apoptotic cells. 

Moreover, the data also indicate that the seeding density of CD34^+^ haematopoietic stem cells is critical and that only in a narrow window reproducible and comparable results can be achieved. At the upper range (high density), the growing colonies might overlap and result in false low values. In the opposite case, low seeding frequencies are more affected by calculation/dilution errors, which lead to false high values. 

When 100/mL to 200/mL CD34^+^ cells are seeded in 24-well plates (250 *μ*L/well), it results in a reproducible ratio of colonies versus seeded CD34^+^ cells. Although the resulting ratio does not necessarily resemble the true ClonE, this might help in comparing quality of different cord blood units.

Regarding the processing-related losses, storage at room temperature for up to 72 h results in higher induction of apoptosis and necrosis in leukocytes (mainly granulocytes). However, in comparison to 4°C, induction of apoptosis in haematopoietic stem cells did not differ significantly. Even long-time storage at 26°C did not seem to affect HSC in a significant manner, while at 37°C it led to immense morphological changes and apoptosis/necrosis in nearly all cells, including CD34^+^ cells, within approximately 40 h. This general loss of viability is in accordance with the data published by Solomon et al. [24], which also observed a similar effect at 37°C after more than 40 hours.

Concerning the impact of dimethyl sulphoxide, the addition of DMSO seems to be the most critical point regarding the loss of viability with an impact stronger than that of the delay between parturition and processing. Apart from a fraction of nonviable HSC already present in the cord blood ahead of processing, the kinetics indicates that a further proportion of the cells become apoptotic within minutes after DMSO is added, even before the final concentration of 10% is reached. Besides this initial loss of viable cells, further induction of apoptosis due to DMSO is much slower. 

Therefore, it can be postulated that CD34^+^ cells contain a fraction that is defined by a higher susceptibility to apoptosis that immediately undergoes apoptosis after exposure to DMSO. Surprisingly, prompt addition of DMSO did not alter the percentage of apoptotic CD34^+^ cells in comparison to slow addition over 10 minutes, although it still has to be confirmed if differences after thawing can be observed between these two methods.

These results demonstrate that Annexin V staining method is a practical and feasible tool for a more precise determination of viability in cord blood transplants. We were able to confirm that current standards regarding the limits for time and temperature are sufficient to warrant CB quality, but additional tests on the impact of DMSO on the apoptosis of HSC are recommended (e.g., modified timescale for addition or final concentration). 

Apart from the Annexin V method, CFU assays can only give reliable results when seeded in a certain range of cell densities, which again proves necessary for individual reproducibility. Here, further improvement might result in CFU assays being finally applicable at least in a semiquantitative manner.

## Figures and Tables

**Table 1:** Ratio of CFU and CD34^+^-cells prior to and after thawing assessed by different methods.

Sample	After volume reduction			After thawing		
	CFU/*µ*L	CD34^+^/*µ*L (ISHAGE)	CD34^+^/*µ*L (AnnV)	CFU/*µ*L	CD34^+^/*µ*L (ISHAGE)	CD34^+^/*µ*L (AnnV)
1	**45**	**29**	24	**33**	**22**	14
2	**46**	**45**	36	**27**	47	**27**
3	**51**	**51**	47	N/A*	N/A*	N/A*
4	**57**	**40**	30	**45**	**20**	12
5	**71**	89	**67**	**50**	78	**51**
6	**75**	111	**76**	**71**	131	**99**
7	**78**	**47**	37	**22**	**24**	13
8	**79**	126	**106**	N/A*	N/A*	N/A*
9	**80**	113	**87**	**39**	60	**34**
10	**96**	115	**100**	**60**	123	**95**

*N/A: not available.

Bold data: results correlating best with the amount of CFU observed assuming a 1 : 1 ratio.
